# The synthetic antihyperlipidemic drug potassium piperate selectively kills breast cancer cells through inhibiting G1-S-phase transition and inducing apoptosis

**DOI:** 10.18632/oncotarget.16872

**Published:** 2017-04-12

**Authors:** Lifei Fan, Xuemin Cao, Huijuan Yan, Qian Wang, Xiaoxia Tian, Lan Zhang, Xiaoyan He, Gereltu Borjihan, Morigen

**Affiliations:** ^1^ School of Life Sciences, Inner Mongolia University, Hohhot 010021, P.R. China; ^2^ Institute of Macromolecular Chemistry and Mongolian Medicine, Inner Mongolia University, Hohhot 010021, P.R. China

**Keywords:** antihyperlipidemic drug, potassium piperate, G1-S-phase transition, apoptosis, breast cancer

## Abstract

*Piper longum* L. is a well-known traditional antihyperlipidemic medicine in China, containing medicinal constituents of piperine, pipernonaline and piperlonguminine in its fruit. However, the antitumor properties of these constituents have not yet been studied. We found that potassium piperate (GBK), a derivative of piperine, inhibited proliferation of cancer cells. GBK selectively inhibited the G1-S-phase transition in breast cancer cells and the G1 arrest was correlated with induction of p27 expression, which is an inhibitor for cyclin-dependent kinases, and inhibition of cyclin A, cyclin E and cyclin B expression. Moreover, GBK treatment led to a downregulation of the mini-chromosome maintenance protein expression and induction of mitochondrial-dependent cell apoptosis in breast cancer cells. Our results also suggested that GBK might also inhibit cancer cell proliferation through epigenetic signaling pathways. A synergistic effect in inhibition of cancer cell proliferation was found when GBK was combined with chemotherapy medicines etoposide phosphate or cisplatin at middle or low doses *in vitro*. These results show that GBK is a novel potential anti-breast cancer drug that inhibits cell proliferation and promotes cell apoptosis.

## INTRODUCTION

Breast cancer is the most common tumor type observed among women worldwide and is the second-leading cause of cancer death in females [1]. Breast cancer is a heterogeneous disease, and the molecular subtypes of breast cancer are classified based on the expression of estrogen receptor (ER), progesterone receptor (PR) and human epidermal growth factor receptor 2 (HER2) [2]. The subtypes are different in their biology, prognostic impact, treatment strategies and pattern of metastasis. Current treatment strategies for breast cancer include adjuvant therapies, such as chemotherapy and endocrine therapy, accompanied with surgical resection [3–6]. ER activation drives breast carcinogenesis in ER-positive breast cancer. Endocrine therapies targeting ER with tamoxifen or aromatase inhibitors are the first-line adjuvant targeted therapies for patients with early stage ER-positive breast cancer [7, 8]. The humanized monoclonal antibody trastuzumab has significantly improved disease-free and overall survival in early stage and advanced HER2-positive breast cancer. Thus, trastuzumab is used as a first-line targeted treatment strategy for HER2-positive breast cancer [9]. There are no recommended targeted therapies for triple-negative breast cancer other than standard chemotherapies, such as anthracyclines and taxanes [8].

The hallmarks of cancer include sustained proliferative signaling, evasion of growth suppressors, resistance to cell death, replicative immortality, angiogenesis, invasion and metastasis, reprogramming of energy metabolism and evasion of immune destruction [10, 11]. Targeting these hallmarks is a rational approach to next-generation cancer therapy. Cisplatin is widely used in advanced breast cancer treatment as a cell cycle phase nonspecific agent [12]. Anthracyclines, e.g., doxorubicin, are a redox-interfering drug to treat solid and non-solid malignancies [13]. Ixabepilone is a new antineoplastic agent that can stabilize microtubule dynamics and lead to apoptotic cell death [14]. Mitoxantrone induces energy imbalance to treat breast cancer [13]. Although effective in tumor therapy, these drugs may lead to side effects, and development of primary and acquired resistance to chemotherapy limit their use in subsequent therapies. Despite the currently available treatment options, breast cancer remains in the top three leading causes of cancer death of women. Thus, there is a great need to develop new therapeutic agents for clinical treatment.

*Piper longum L*. is a well-known traditional antihyperlipidemic medicine in China that contains medicinal constituents of piperine (GBO), pipernonaline and piperlonguminine (GBN) in its fruit [15]. These constituents exhibit antihyperlipidemic activity, which is comparable to that of the commercial antihyperlipidemic drug simvastatin [15]. Extensive study of GBN validates that it has high antihyperlipidemic activity and low cytotoxicity [16]. During the synthesis procedure of GBN, there is an intermediate product potassium piperate (GBK) that also shows antihyperlipidemic activity but is a hydrophilic compound relative to lipophilic GBO (unpublished data). Another report showed that Piperlongumine, which is also a natural product isolated from the plant species *Piper longum L*., can increase the level of reactive oxygen species (ROS) and apoptotic cell death of cancer cells, but it has little effect on either rapidly or slowly dividing primary normal cells [17]. Piplartine, an alkaloid/amide component of the *Piper* species, induces inhibition of leukemia cell proliferation, triggering both apoptosis and necrosis pathways [18]. However, the anti-cancer properties of GBK have not been explored yet. In this study, we aim to characterize the effects of GBK on breast cancer and elucidate the underlying molecular mechanism responsible for proliferation inhibition.

## RESULTS

### Selective killing effect of GBK in cancer cells

**Figure 1:**
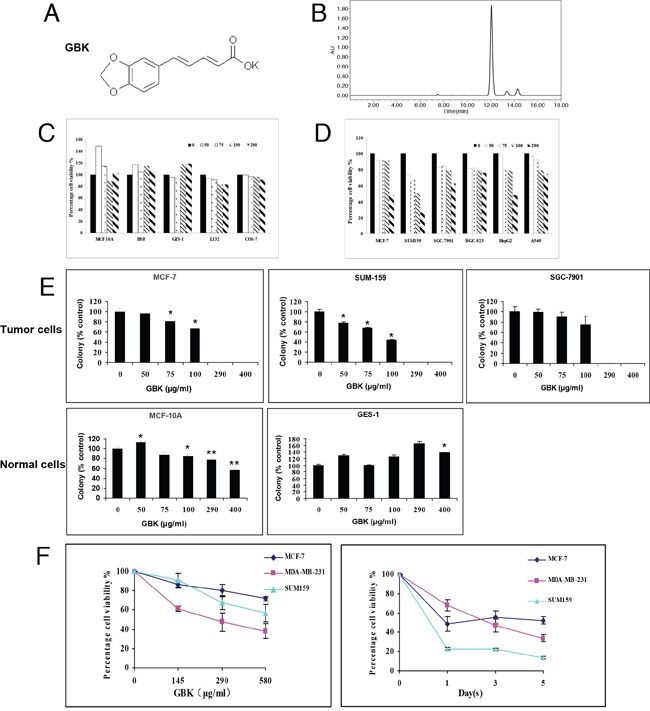
Selective killing effect of GBK in cancer cells **(A)** Chemical structure of GBK. **(B)** The purity of synthesized GBK was measured by high-performance liquid chromatography (HPLC). The sample of GBK had only one sharp peak at 12 min as a retention time on the HPLC chromatogram. GBK was HPLC-purified (~99% purity) before the treatment. **(C, D)** Normal human cells, including human mammary epithelial cells (MCF-10A), human skin fibroblast cells (HSF), human gastric mucosa cells (GES-1), and human lung epithelial cells (L132), African green monkey kidney cells (COS-7), and human cancer cell lines, including human mammary cancer cells (MCF-7 and SUM159), human gastric cancer cells (SGC-7901 and BGC-823), human liver cancer cells (HepG2) and human lung cancer cells (A549), were grown in 96-well plates and treated with GBK at 0–290 μg/ml for 48 h. Cell viability was measured by CCK-8 assay. **(E)** Normal and tumor cells were treated with GBK at 0–400 μg/ml for 14 days, and live cells were stained by crystal violet. ddH_2_O was used as control. Columns show data expressed as means ± standard deviation (SD) of three independent experiments. *P < 0.05; **P < 0.01. **(F)** Cell viability of three human breast cancer cell lines treated with GBK was measured by CCK-8 assay. Independent experiments were repeated in triplicate; bars, SDs.

The anti-cancer effects of GBK, a derivative of piperine, have not been previously investigated. We thus examined the effects of GBK on the viability of cultured cancer cells and normal cells (Figure 1C and 1D). The IC_50_ values of GBK in various human cancer cell lines and normal cell lines were determined by CCK-8 assay (Supplementary Table 1). Cultured normal cell lines (MCF-10A, HSF, GES-1, L132 and COS-7) and human cancer cell lines (MCF-7, SUM-159, SGC-7901, BGC-823, HepG2, and A549) were grown in 96-well plates and treated with GBK at 0 to 290 μg/ml for 48 h. Cell viability was then measured by CCK-8 assay. GBK treatment markedly increased cell death in cancer cells but not in normal cells, indicating that GBK exhibits a cancer cell-selective killing property.

To determine whether GBK inhibits anchorage-dependent growth, we performed colony formation assays. MCF-7, SUM-159, SGC-7901, MCF-10A and GES-1 cells were treated with GBK at 0–400 μg/ml concentrations for 14 days, and the colony formation capacity was determined by counting the number of colonies stained by crystal violet. GBK exhibited cytotoxicity only in tumor cells (MCF-7, SUM159 and SGC-7901) and not in normal human breast epithelial cells (MCF-10A) or human gastric mucosa cells (GES-1) at less than 290 μg/ml. At higher concentration of GBK (400 μg/ml), slight cytotoxicity was observed in MCF-10A normal human breast epithelial cells. Notably, GBK was effective in killing cancer cells at concentrations less than 100 μg/ml (Figure 1E and Supplementary Figure 1).

We next further investigated whether GBK affects cellular proliferation of human cancer cells. We analyzed the effects of GBK on the proliferation of three breast cancer cell lines (MCF-7, MDA-MB-231 and SUM-159) in dose-dependent and time-dependent experiments. Cell viability was measured by CCK-8 analysis. Treatment of three different breast cancer cell lines with 0 to 580 μg/ml GBK for 48 h revealed a dose-dependent decrease in cell proliferation (Figure 1F). We also observed inhibition of proliferation of cells incubated with 290 μg/ml (IC_50_ of MCF-7) GBK for 0, 1, 3 and 5 days in a time-dependent manner (Figure 1F).

### GBK selectively inhibits the G1-S-phase transition of MCF-7 cells

**Figure 2:**
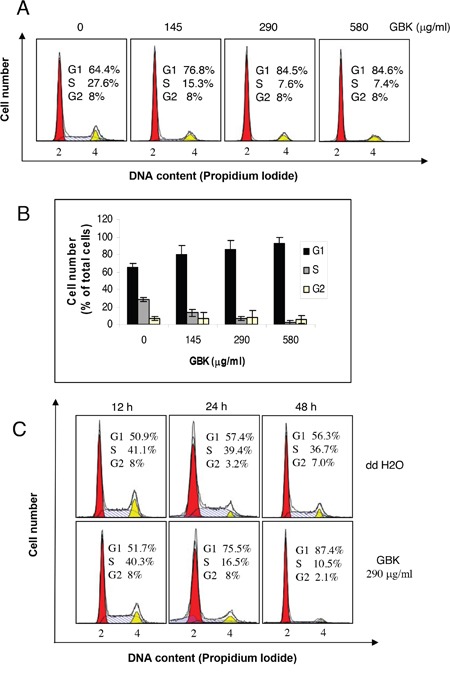
Induction of G1 arrest in GBK-treated MCF-7 breast cancer cells **(A, B)** Proliferating MCF-7 cells were treated with different concentrations of GBK (0–580 μg/ml) for 48 h and cell cycle distributions were analyzed by flow cytometry. Representative flow cytometry data is shown in **(A)**, and columns in **(B)** show the data expressed as means ± SD of three independent experiments. **(C)** Proliferating MCF-7 cells were treated with 290 μg/ml GBK for the indicated time and cell cycle distributions were analyzed by flow cytometry. ddH_2_O was used as control.

To determine whether the growth inhibition of cancer cells by GBK was caused by cell cycle arrest, cells were treated with various concentrations of GBK for 48 h and cell cycle distributions were analyzed by flow cytometry. We found that upon exposure to increased concentrations of GBK, only the breast cancer cell line MCF-7 showed a G1 phase arrest accompanied by a decrease in S phase compared with untreated control cells (Figure 2A and 2B, Supplementary Figure 2). We further investigated the effects of GBK on MCF-7 cell cycle progression in time course experiments. Proliferating MCF-7 cells were treated with 290 μg/ml GBK for 12, 24 or 48 h. We observed an increase in G1 phase cells in GBK-treated MCF-7 cells relative to the control groups receiving no GBK (Figure 2C). In parallel, there was a reduction in the percentage of S phase cells. Taken together, these results demonstrate that GBK selectively inhibits the G1-S-phase transition and causes a G1 cell cycle arrest in the MCF-7 breast cancer cell line.

### GBK activates sets of genes in response to replication stress in MCF-7 cells

**Figure 3:**
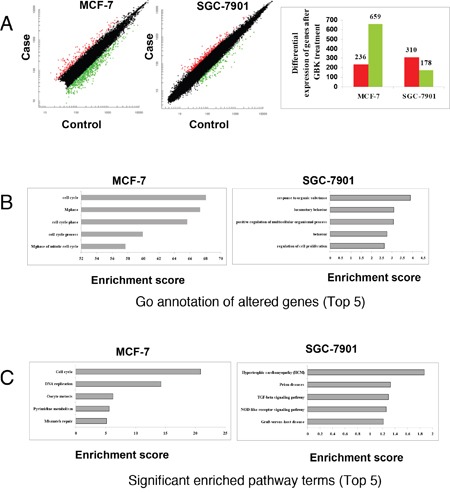
Comparison of transcriptomic responses to GBK treatment between MCF-7 and SGC-7901 cells **(A)** mRNA isolated from GBK-treated (1.5 fold IC_50_ for 48 h) MCF-7 and SGC-7901 cells was subjected to DNA microarray analysis. The scatter plots show the deregulated genes in case versus control, and the bar plots show the number of differential expressed genes in case versus control. Red and green indicate the upregulated and downregulated genes, respectively. Fold change filters required upregulated genes to be present at levels at least 2-fold of controls, and for downregulated genes to be 0.5-fold lower than of controls. **(B)** The bar plots show the top five gene ontology terms of altered genes in GBK-treated (1.5 fold IC_50_ for 48 h) MCF-7 and SGC-7901 cells. **(C)** The bar plots show the top five significant enriched pathway terms in GBK-treated (1.5 fold IC_50_ for 48 h) MCF-7 and SGC-7901 cells.

To investigate the molecular mechanisms by which GBK selectively blocks cell cycle progression in breast cancer cells, we carried out a microarray analysis following GBK treatment and examined regulatory differences between GBK-sensitive (MCF-7) and GBK-insensitive (SGC-7901) cells. We compared the gene expression patterns between MCF-7 and SGC-7901 cells in the presence or absence of GBK treatment (GBK concentration used in the assay is 1.5 fold IC_50_ of MCF-7 and SGC-7901 cells). The results showed that 236 genes were upregulated (>2-fold) and 659 genes were downregulated (< 0.5-fold) in MCF-7 cells treated with GBK for 48 h. In SGC-7901 cells, the results revealed 310 upregulated (>2-fold) and 178 downregulated (< 0.5-fold) genes after GBK treatment (Figure 3A).

Further analyses revealed that GBK stimulated different sets of genes between MCF-7 and SGC-7901 cells (Figure 3B and 3C, Supplementary Table 2 and Supplementary Table 3). In particular, GBK treatment altered the expression of genes involved in DNA replication and cell cycle in MCF-7 cells (Figure 3B and 3C, Supplementary Table 2). In contrast, GBK did not affect these genes in SGC-7901 cells and instead affected genes in steroid hormone biosynthesis signaling pathways (Figure 3B and 3C, Supplementary Table 3). These data indicate that GBK treatment mainly interferes with DNA replication and cell cycle signaling in MCF-7 cells.

**Figure 4:**
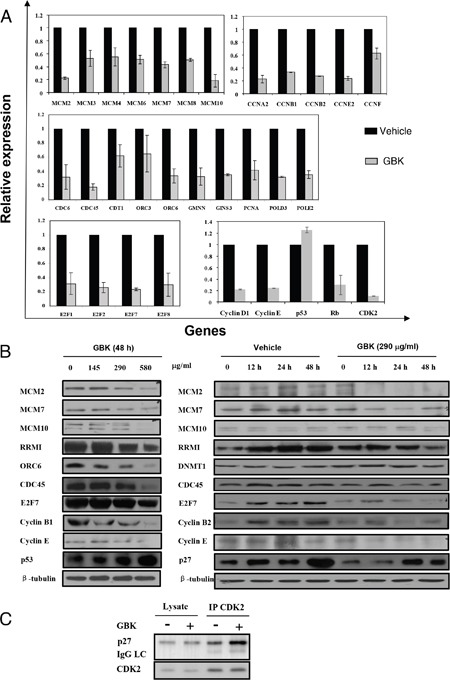
GBK induces cell cycle arrest at G1/S phase by downregulation of RNA and proteins important for G1-S phase transition in cancer cells **(A)** Differential expression of genes in MCF-7 cells after treatment with GBK was analyzed by RT-qPCR. Genes related to replication and cell cycle were downregulated, while the tumor repressor gene *p53* was upregulated. **(B)** Effect of GBK on the expression of cell cycle-related proteins in MCF-7 cells in dose-dependent and time-dependent experiments. MCF-7 cells were treated with various concentrations of GBK (0–580 μg/ml) for 48 h, or 290 μg/ml GBK in a time course. Protein lysates were analyzed by western blot. β-tubulin was used as an internal control. **(C)** Effect of GBK on G1 cell cycle regulatory complex formation. MCF-7 cells were treated with 290 μg/ml of GBK for 48 h. Total cell lysates were immunoprecipitated with anti-CDK2 antibody and analyzed by western blot analysis using anti-p27 and anti-CDK2 antibodies.

To verify the microarray data showing that the most differentially expressed genes in MCF-7 cells after GBK treatment are related with DNA replication and cell cycle progression, we performed RT-qPCR using the same total RNA prepared for the microarray (Figure 4A and Supplementary Figure 3). Among deregulated genes, the genes related with DNA replication and cell cycle were selected. For instance, we examined the MCM protein family that is essential for initiation and elongation of DNA replication in all eukaryotes (MCM2, MCM3, MCM4, MCM6, MCM7, MCM8 and MCM10), cyclins and cyclin dependent kinases (CDKs) that play critical roles in the control of cell cycle progression (cyclin A2, cyclin B1, cyclin B2, cyclin E2 and cyclin F), the components of pre-replication complex (pre-RC) and the proteins involved in activation of the pre-RC and initiation of replication (CDC6, CDC45, CDT1, ORC3, ORC6, GMNN, GINS3, PCNA, POLD2 and POLD3), and the E2F family transcriptional factors that trigger G1 cell cycle progression (E2F1, E2F2, E2F7 and E2F8). All these genes showed a tendency towards decreased expression after GBK treatment, consistent with the results from the transcriptional microarray analysis, suggesting that the microarray data is reliable. Specifically, we observed a > 2-fold downregulation in expression of cyclin E and cyclin A (cyclin E and CDK2 interact during the G1 to S transition, CDK2-cyclin A regulates S phase progression), MCM2 and MCM10 (MCM2-7 complex serves as the DNA helicase during replication, MCM10 may serve as the coordinator for MCM2-7 helicase and DNA polymerase, facilitating their roles during replication initiation and elongation), ORC6 and CDC45 (ORC is the eukaryotic origin recognition complex, and the CDC45-MCM2-7-GINS complex acts as the active replicative helicase), and E2F7 (E2F family transcriptional factors stimulate the expression of proteins required in S phase). In addition, we speculated that the genes in the same signaling pathways as the identified deregulated genes should also show differential expression after GBK treatment, even though they were not selected by microarray analysis. We selected the tumor suppressor gene *p53* and *Rb* and cell cycle-related genes *cyclin D*, *cyclin E* and *CDK2* for RT-qPCR, and found that *p53* was upregulated and *Rb, cyclin D*, *cyclin E* and *CDK2* were downregulated after GBK treatment.

We also investigated whether the expression level of cell cycle and DNA replication-related proteins was altered with GBK treatment. MCF-7 cells were incubated with various concentrations of GBK for 48 h and examined by western blot analysis. We observed a dose-dependent activation of the tumor suppressor p53 as well as decreased expression of G1/S cell cycle-regulating factors cyclin E, cyclin B1, E2F7, and DNA replication-related proteins MCMs, CDC45, ORC6, and ribonucleotide reductase M1 (RRMI) in MCF-7 cells after exposure to increasing concentrations of GBK (Figure 4B). In addition, the levels of the CDK inhibitor p27 were induced by GBK treatment in a time-dependent manner, while MCMs and cyclin E showed a time-dependent decrease in expression level (Figure 4B). Furthermore, immunoprecipitation analysis revealed that GBK-treated cells showed an increase in the binding of p27 to the cyclin E/CDK2 complex compared to untreated control (Figure 4C), thus inhibiting releasing E2F transcription factor to trigger G1 cell cycle progression. These data reveal that the inhibition of breast cancer cell proliferation by GBK is associated with the induction of G1/S phase arrest.

We proposed that the anti-cancer mechanism of GBK in MCF-7 cells is by induction of G1 arrest. GBK treatment would somehow propagate the signal via a downstream substrate such as p53, which would in turn activate the transcription of CDK inhibitor p27, suppress the activities of G1/S-CDK complexes (cyclin E/CDK2), inhibit the phosphorylation of Rb protein, restrict the release of Rb from E2F, and lead to downregulation of MCM transcription and replicative arrest.

### GBK induces MCF-7 cell apoptosis

**Figure 5:**
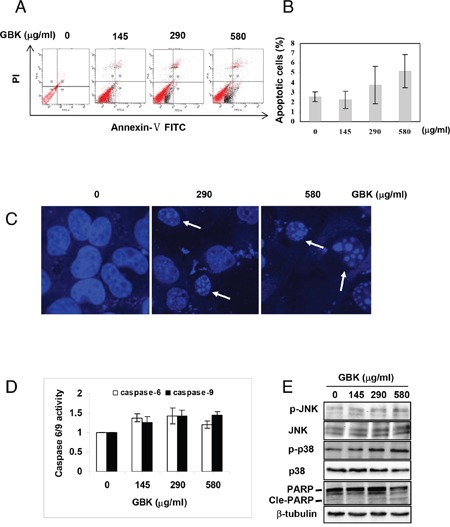
GBK induces apoptosis in MCF-7 breast cancer cells **(A, B)** Induction of apoptosis in human MCF-7 breast cancer cells was determined by flow cytometry after treatment with GBK (0–580 μg/ml) for 48 h. Representative flow cytometry data is shown in **(A)**, and columns in **(B)** show the data expressed as means ± SD of three independent experiments. **(C)** GBK treatment induced apoptotic morphology in MCF-7 cells. MCF-7 cells were treated with GBK (0–580 μg/ml) for 48 h. Cell morphology was observed using a confocal microscope (Zeiss) after Hoechst 33258 staining. White arrows indicate the fragmented DNA in apoptotic cells. **(D)** GBK induced a dose-dependent activation of caspase 6 and caspase 9. MCF-7 cells were treated with GBK (0–580 μg/ml) for 48 h, and the activations of caspase 6 and caspase 9 were measured by ELISA. Columns show the data expressed as means ± SD of three independent experiments. **(E)** After treated with 0–580 μg/ml GBK for 24 h, the phosphorylation status of JNK (or p38 MAPK) and the apoptosis-associated protein expression of cleave PARP were detected using western blot. β-tubulin was used as internal control.

We further examined the cancer cell apoptosis induced by GBK using Annexin V/propidium iodide (PI) staining and flow cytometry analysis. MCF-7 breast cancer cells showed a concentration-dependent increase in apoptosis in response to GBK exposure (Figure 5A and 5B). Morphology analysis confirmed that MCF-7 cells exhibited apoptotic characteristics after treatment with GBK for 48 h, with apoptotic nuclear morphology observed with Hoechst 33258 staining, such as nuclear condensation and fragmentation (Figure 5C). Treatment of MCF-7 cells with 0–580 μg/ml GBK for 48 h also resulted in a dose-dependent activation of caspase 6 and caspase 9 by ELISA (Figure 5D).

Previous studies have demonstrated that MAPK signaling pathways are central regulators of cell growth and apoptosis in response to stress conditions [19]. ERK is a mitogen-activated proliferation factor, whereas JNK and p38 MAPK play key roles in crosstalk between apoptosis and autophagy induced by genotoxic stress [20]. We found that stimulation of MCF-7 cells with GBK induced an increase in phosphorylation of JNK and p38 MAPK in a dose-dependent manner (Figure 5E). Consistently, GBK dose-dependently increased the expression of Cle-PARP. These results indicated that GBK induced cell apoptosis in MCF-7 cells through activation of JNK and p38 MAPK pathways.

Our results revealed that the mechanism by which GBK induced apoptosis is via activating the caspase 6 and caspase 9-dependent pathway, and that p38/JNK MAPK signaling pathways also play a crucial role in the GBK-induced apoptosis. These results suggest that the anti-cancer effect of GBK is also associated with the induction of cell apoptosis.

### GBK induced cytotoxicity in MCF-7 cells is independent of intracellular ROS generation

**Figure 6:**
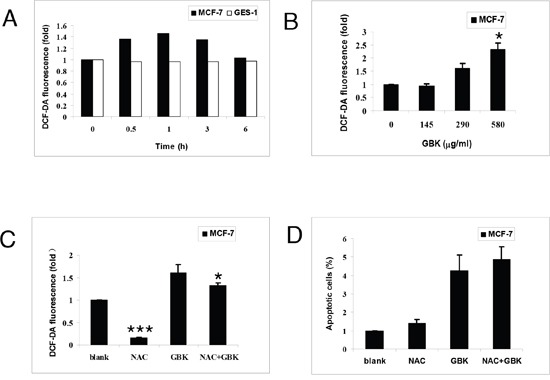
GBK enhances ROS accumulation in breast cancer cells **(A)** Intracellular ROS generation induced by 290 μg/ml GBK treatment for 0–6 h in MCF-7 and GES-1 cells was measured by incubating with DCFH-DA (10 μM) and followed by flow cytometry analysis. **(B)** Intracellular ROS generation induced by increasing doses of GBK (0–580 μg/ml for 0.5 h) was measured in MCF-7 cells by incubating with DCFH-DA (10 μM) and flow cytometry analysis. **(C)** MCF-7 cells were pre-incubated with or without 5 mM NAC for 2 h before exposure to GBK (290 μg/ml) for 0.5 h. Intracellular ROS generation was measured by flow cytometry. **(D)** MCF-7 cells were pre-incubated with or without 5 mM NAC for 2 h before exposure to GBK (290 μg/ml) for 24 h. Percentage of cell apoptosis was determined by Annexin-V/PI staining and flow cytometry. Columns show the data expressed as means ± SD of three independent experiments. *P < 0.05; ***P < 0.001.

Normal cells have low basal levels of ROS and therefore a diminished reliance on the ROS stress response pathway, whereas cancer cells have high levels of ROS and might therefore be expected to have a strong reliance on the ROS stress response pathway [17]. Thus, perturbing redox and ROS homeostasis by therapeutic agents is a promising strategy for cancer treatment [17, 21]. We next determined the effect of GBK on cellular ROS levels in MCF-7 human breast cancer cells through flow cytometry using the redox-sensitive fluorescent probe 2’ -, 7’-dichlorofluorescein diacetate (DCFH-DA). Treatment with 290 μg/ml GBK for 0.5 h to 3 h caused a marked increase in ROS levels in MCF-7 cells relative to normal cells (Figure 6A). Moreover, treatment of MCF-7 cells with GBK also resulted in a dose-dependent increase in ROS levels (Figure 6B). Co-treatment with NAC partially reversed the GBK-induced increase in ROS but had no effect on GBK-induced cell death (Figure 6C and 6D). In contrast to the results in MCF-7 cells, GBK did not cause an increase in ROS levels in human gastric mucosa cells GES-1 (Figure 6A), indicating a selective induction of ROS in breast cancer cells. These results revealed that GBK could mediate ROS elevation in MCF-7 cells, but GBK-induced apoptosis in MCF-7 cells is independent of intracellular ROS generation

### *In vivo* antitumor effects of GBK on NOD/SCID mice bearing MCF-7 xenografts

**Figure 7:**
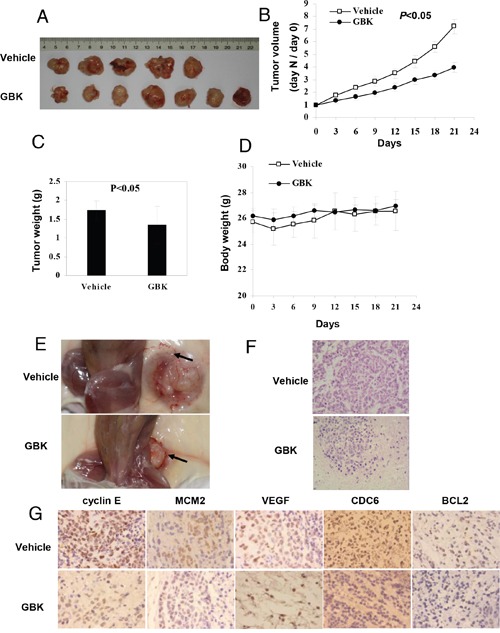
*In vivo* antitumor effects of GBK on NOD/SCID mice bearing MCF-7 cells **(A)** MCF-7 cells were injected into flanks of NOD/SCID mice, and when tumors had grown to ~5–6 mm in diameter, GBK (10 mg/kg) or physiological saline (0.9% m/v) was administered intraperitoneally daily for 21 days. **(B)** On day 0, the tumor size was normalized to 1 for all the groups. Tumor volume was monitored and measured once every 3 days. Error bars represent the standard error of the mean (S.E.M.) *P* < 0.05 versus control group. **(C)** On day 21, tumors were excised and subjected to weight analysis. Error bars represent the S.E.M. *P* < 0.05 versus control group. **(D)** Body weight of the NOD/SCID mice in vehicle-treated and GBK-treated mice. Error bars represent the S.E.M. **(E)** Arrows show that GBK treatment inhibited the formation of blood vessels in xenograft-tumor mice. **(F)** Histological morphology of tumor tissue sections from MCF-7 tumor-bearing mice treated with GBK or 0.9% physiological saline after 21 days, stained with hematoxylin and eosin. **(G)** Tumor tissue sections were subjected to immunohistochemistry detection using the indicated antibodies.

To evaluate the role of GBK in controlling mammary tumor growth *in vivo*, we examined the ability of GBK to suppress the growth of MCF-7 xenografts in NOD/SCID mice. When tumor sizes had grown to about 5–6 mm in diameter in NOD/SCID mice bearing MCF-7 xenografts, GBK (10 mg/kg) or physiological saline (0.9% m/v) was administered intraperitoneally daily for 21 days and notable anti-tumor effects were observed. Treatment with GBK resulted in a significant reduction in tumor volume compared to that observed in the vehicle group (*P* < 0.05) (Figure 7A and 7B). The tumor weight was decreased by GBK administration at the dosage of 10 mg/kg (Figure 7C). There was no obvious change in the body weights of the mice during treatment (Figure 7D). In addition, GBK treatment inhibited the formation of blood vessels in xenograft-tumor mice (Figure 7E).

At day 21, mice were euthanized and tumors were excised and processed for histological examination. The vehicle-treated control mice showed severe malignant progression. In contrast, the tissues from GBK-treated mice showed a hyperplastic-like, non-malignant phenotype (Figure 7F). Immunohistochemical stainings of cyclin E, MCM2, VEGF, CDC6 and BCL2 were performed in tumor tissues, and the staining intensities of cyclin E, MCM2, CDC6 and BCL2 were decreased in the GBK treatment group in xenograft-tumor mice compared to the control group (Figure 7G). The vehicle-treated tumor sections showed high expression of VEGF, but no obvious level of staining was observed in the GBK-treated tissue sections (Figure 7G). These results revealed a potent inhibitory effect of GBK on MCF-7 tumor growth *in vivo*.

### Synergistic antitumor effect of GBK with cisplatin or etoposide phosphate

To further investigate whether GBK could be used in clinical treatment for breast cancers, we tested the antitumor effect of GBK when used in combination with cisplatin (DDP) or etoposide phosphate (VP-16). MCF-7 cells were treated with different concentrations of GBK, VP-16 or DDP, either alone or in combination, for 48 h and cytotoxicity effects were measured by CCK-8 assays. MCF-7 cells treated with GBK (0–100 μg/ml), VP-16 (0–100 μg/ml), or DDP (0–20 μg/ml) for 48 h all showed inhibition of cell proliferation in a dose-dependent manner (Tables 1, 2 and 3). The IC_50_ values of GBK, VP-16 or DDP in MCF-7 cells were 290 μg/ml, 8.37 μg/ml, and 5 μg/ml, respectively. Notably, the inhibition of cell proliferation in the GBK+VP-16 group and the GBK+DDP group was higher than those in the single drug groups (Tables 4 and 5). Furthermore, GBK combined with VP-16 or DDP at middle or low doses (< IC_50_) showed a synergistic effect (Table 4: lines 2 and 4; Table 5: lines 2, 4 and 5). Together these data indicate that when VP-16 or DDP was used in combination with GBK, the same proliferation inhibition effect could be achieved with reduced amount of VP-16 or DDP used. These results demonstrate that GBK is a novel potential clinical anti-breast cancer drug with mild toxicity and excellent efficacy in combination regimens.

**Table 1:** Inhibitory effect of breast cancer cell line MCF-7 treated with GBK

Groups	Dosage (g/ml)	N	OD value	Inhibitory rate (%)
control	0	3	0.8440±0.14009	-
	1	3	0.8025±0.10363	4.92
	2	3	0.7600±0.12529	9.95
	5	3	0.7145±0.10652	15.34
GBK	10	3	0.7075±0.11114	16.17
	20	3	0.6755±0.11114	19.96
	40	3	0.6720±0.07217	20.38
	100	3	0.6305±0.09267	25.30

**Table 2:** Inhibitory effect of breast cancer cell line MCF-7 treated with VP-16

Groups	Dosage (g/ml)	N	OD value	Inhibitory rate (%)
control	0	3	1.2050±0.12298	-
	1	3	1.1806±0.14440	2.15
	2	3	1.1602±0.11280	3.63
	5	3	0.7684±0.19929	35.53
VP-16	10	3	0.4827±0.02650	59.72
	20	3	0.1124±0.10042	90.36
	40	3	0.1062±0.00449	91.11
	100	3	0.0554±0.01161	95.43

**Table 3:** Inhibitory effect of breast cancer cell line MCF-7 treated with DDP

Groups	Dosage (g/ml)	N	OD value	Inhibitory rate (%)
control	0	3	0.7528±0.03761	-
	0.5	3	0.6349±0.02314	15.66
	1	3	0.5794±0.02280	23.03
	2	3	0.4163±0.01411	44.70
DDP	5	3	0.3759±0.01405	50.07
	10	3	0.3166±0.00421	57.94
	20	3	0.2109±0.00959	71.98

**Table 4:** Inhibitory effect of breast cancer cell line MCF-7 treated with GBK and VP-16

Groups	Dosage (g/ml)	N	OD value	Inhibitory rate (%)
control	0	3	0.6217±0.06014	-
	1+1	3	0.3863±0.01137	37.34
	1+10	3	0.2243±0.05315	63.14
GBK+VP-16	5+5	3	0.3303±0.06461	45.96
	5+10	3	0.2180±0.03318	64.35
	10+10	3	0.1963±0.02108	68.06

**Table 5:** Inhibitory effect of breast cancer cell line MCF-7 treated with GBK and DDP

Groups	Dosage (g/ml)	N	OD value	Inhibitory rate (%)
control	0	3	1.2697±0.05868	-
	1+1	3	0.5672±0.12378	55.33
	1+5	3	0.0882±0.00375	93.05
	5+1	3	0.4545±0.09009	64.20
GBK+DDP	2+2	3	0.1317±0.06300	89.63
	2+5	3	0.0547±0.01750	95.69
	5+5	3	0.0218±0.01371	98.28

## DISCUSSION

**Figure 8:**
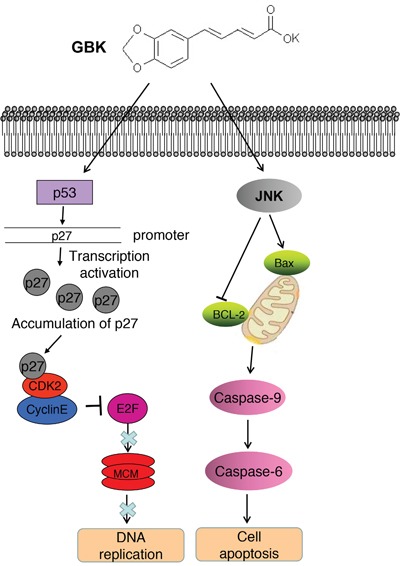
The model for the role of GBK in inhibiting breast tumorigenesis GBK selectively inhibits the G1-S-phase transition in breast cancer cells by induction of the CDK inhibitor p27 expression as well as inhibition of cyclin E/CDK2 complex activities. In turn, MCM proteins are markedly downregulated in MCF-7 cells treated with GBK. GBK also kills breast cancer cells by inducing cell apoptosis through JNK signaling.

In this study, we investigated the potential ability of the synthetic antihyperlipidemic drug GBK for cancer treatment. Our results indicate that GBK selectively kills breast cancer cells through inhibiting the G1-S-phase transition and inducing apoptosis. These findings support the further development of GBK for antitumor therapies. The model for the role of GBK in inhibiting breast tumorigenesis is illustrated in Figure 8.

Sustained proliferative signaling is one of the hallmarks of cancer. The MCM protein family is essential for DNA replication in all eukaryotes, and thus anti-MCM molecules can be used as potential anti-cancer drugs [22]. We demonstrated in this paper that GBK selectively inhibited the G1-S-phase transition in breast cancer cells and the G1 arrest was correlated with induction of p27 expression, which is an inhibitor for cyclin-dependent kinases, and inhibition of cyclin A, cyclin E and cyclin B expression. Moreover, GBK treatment led to a downregulation of the mini-chromosome maintenance protein expression. Progression from the G1 phase to the S phase of the cell cycle is stimulated by cyclin D-CDK4/6 and cyclin E-CDK2, followed by phosphorylation and inactivation of pRB, which releases the E2F transcriptional factor and enables cells to progress through the G1/S checkpoint and commit to DNA synthesis [23, 24]. Interestingly, we found the DNA replication negative regulator geminin was also downregulated in GBK-treated MCF-7 cells (Figure 4A). A previous study reported that suppression of geminin activity could selectively kill cancer cells by inducing DNA re-replication and DNA damage that spontaneously triggered apoptosis [25]. Thus, we propose that one of the anti-tumor mechanisms of GBK treatment would somehow propagate the signal via a downstream substrate such as p53, which would in turn activate the transcription of the CDK inhibitor p27 and increase the binding of p27 to CDK2, suppress the activities of G1/S-CDK (cyclin E/CDK2), inhibit the phosphorylation of Rb protein, restrict the release of Rb from E2F, and lead to downregulation of MCM transcription and replicative arrest.

Resisting cell death is another hallmark of cancer. Several pro-apoptotic genes, such as BMF, BAX and NOXA genes, were upregulated after GBK treatment in MCF-7 cells while pro-survival genes (such as BCL-2 and BIRC5 genes) showed reduced mRNA levels (Supplementary Figure 3A and Supplementary Table 5). JNK is an important signaling factor in triggering the mitochondria-mediated apoptotic pathway in cancer cells [26–28]. We found that the enzymatic activity of caspase 6 and caspase 9 were increased in MCF-7 cells treated with GBK. JNK was also phosphorylated after GBK treatment. We thus concluded that when MCF-7 cells were treated with GBK, the JNK-induced mitochondrial apoptotic pathway was activated.

Aberrant DNA methylation frequently occurs in carcinogenesis [29, 30]. Dysregulation of tumor DNA methylation includes global hypomethylation and hypermethylation of CpG islands at the promoters of tumor suppressor genes, both of which have been observed in almost every cancer type [31, 32]. Aberrant DNA methylation is also an important mechanism in breast carcinogenesis, such as methylation in the promoter region of the tumor suppressor gene *p53* [33, 34] or the homologous DNA recombination repair related gene *BRCA1* [35, 36], as well as the E-cadherin promoter DNA methylation-induced loss of E-cadherin expression in basal-like breast cancer [37]. In addition, histone lysine methylation has emerged as an important modification due to its central role in transcriptional regulation. Loss of H3K4 methylation (trimethylation of histone H3 at lysine 4) and gain of H3K9 methylation (dimethylation of histone H3 at lysine 9) are most closely related with DNA methylation. DNMT1, DNMT3A, and DNMT3B all belong to a family of highly related DNA methyltransferase enzymes that transfer a methyl group to the cytosine in a CpG dinucleotide, which commonly occurs in the promoter region of genes [38]. SUV39H1 is a key component of the methyltransferase responsible for H3K9me3 [39]. We demonstrated here that the transcription of DNA methyltransferases DNMT1, DNMT3A, DNMT3B and COQ3 genes as well as the histone methyltransferase DOT1, SUV39H1 and SUV39H2 were downregulated in GBK-treated MCF-7 cells (Supplementary Figure 3B), indicating that GBK could inhibit breast cancer progression by reducing the DNA methylation level in the promoters of tumor suppressor genes or the global genome. Therefore, future work will be needed to reveal how these epigenetic regulations are achieved by GBK treatment.

Over the past decades, there has been growing interest in developing natural products and their derivatives as potential therapeutic applications for malignant diseases [40]. Natural products and their derivatives can also be used as co-adjuvants of classic chemotherapeutic drugs to increase their effectiveness [41–43]. In this study, we demonstrated synergistic cytotoxic effects when GBK was combined with VP-16 or DDP at middle or low doses through inhibiting cell proliferation. These results suggest that the combination of GBK and VP-16/DPP may be a promising therapeutic option for breast cancer. Further investigations are needed to test the combination therapy of GBK along with standard chemotherapeutic agents in clinical trials.

Alterations of lipid metabolism have been increasingly regarded as a hallmark of cancer cells, as the mevalonate pathway for cholesterol biosynthesis and protein prenylation has been implicated in many aspects of tumorigenesis. The oncoproteins like Ras family members (Ras and Rho GTPases) undergo post-translational isoprenylation for their activity [44]. Antihyperlipidemic drugs, such as statins, inhibit oncoprotein prenylation through isoprenoiding intermediates farnesyl pyrophosphate (FPP) and geranyl pyrophosphate (GPP), which are essential for modification of Ras family members that bind to FPP and/or GPP through isoprenylation [45]. Thus, reduction in isoprenoids (FPP and GPP) could inhibit tumor cell proliferation, enhance tumor apoptosis, inhibit angiogenesis, migration, and invasion, and impair metastasis [46]. Accumulating preclinical data suggest that statins may have chemopreventative potential against several types of cancer, mostly gastrointestinal tumors, breast cancer and prostate cancer [47]. The synthetic drug GBK, which also shows antihyperlipidemic activity, is an attractive potent therapeutic agent to treat cancer, as it could be used by large populations to treat non-cancer-related medical situations. Further preclinical data, such as the starting time of GBK therapy, duration and dose, are required to validate the effect of GBK in prevention and treatment of breast cancer when used as a daily drug.

In summary, we demonstrated that GBK is a multi-targeting anti-breast cancer compound. The MCF-7 cell system is one of the most recognized models for estrogen receptor (ER)-positive breast cancer [48]. We propose a model in which after GBK treatment, MCF-7 cell proliferation is inhibited by G1-S-phase arrest and JNK signaling pathway is also induced to selectively kill breast cancer cells (Figure 8). A synergistic antitumor effect was observed when GBK was used in combination with classical chemotherapeutic drugs (VP-16 or DDP). In addition, *in vivo* studies highlighted the anti-breast carcinogenesis behavior of GBK. Therefore, our findings reveal the potential of the synthetic antihyperlipidemic drug GBK in clinical applications for breast cancer and offer a novel treatment strategy against metastatic breast cancer.

## MATERIALS AND METHODS

### Ethics statement

Investigation has been conducted in accordance with the ethical standards and according to the Declaration of Helsinki and according to national and international guidelines. All experiments adhered with the National Research Council Guide for the Care and Use of Laboratory Animals and were approved by the Institutional Animal Care and Use Committee at Inner Mongolia University.

### Chemicals and reagents

GBK (purity >98%) (Figure 1A and 1B) was kindly provided by Professor Gereltu Borjihan (Institute of Macromolecular Chemistry and Mongolian Medicine, Inner Mongolia University). GBK is an intermediate product during the chemical synthesis procedure of GBN. Briefly, piperine (10 g) was dissolved in 300 ml of anhydrous ethanol containing 20% KOH (wt %) in a 500 ml reaction flask equipped with a reflux condenser. The mixture was refluxed for 10 h to precipitate GBK. After filtration, the precipitate was washed three times with anhydrous ethanol [49]. The drug was dissolved in distilled water to make a 10 mg/ml stock solution, which was further diluted to the appropriate concentration with culture medium before each experiment. The IC_50_ of GBK in MCF-7 cells is 290 μg/ml (1.16 mM). Control experiments contained only distilled water. Distilled water was prepared with a Milli-Q water purification system from Millipore (Molsheim, France).

Monoclonal antibodies to RRM1 and MCM7 were from ProteinTech (Chicago, Illinois, USA) and Abcam (Cambridge, UK), respectively. Mouse anti-tubulin antibody was from TransGen Biotechnology (Beijing, China). Polyclonal antibodies to cleaved PARP, CDK2, E2F7, CDC45, DNMT1, cyclin B1, cyclin B2, p53, pRb, JNK, p-JNK, p38, p-p38, ORC6, VEGF, CDC6, BCL2 and MCM10 were from ProteinTech. Polyclonal antibody to MCM2 was from Abcam. Polyclonal antibodies to cyclin E and p27 were from Santa Cruz Biotechnology (Santa Cruz, California, USA). Goat anti-Mouse IgG (H+L) HRP Conjugate Secondary Antibody and Goat anti-Rabbit IgG (H+L) HRP Conjugate Secondary Antibody were obtained from TransGen Biotechnology.

The FITC-conjugated Annexin V Apoptosis Detection Kit and propidium iodide (PI) were purchased from BD Pharmingen (Franklin Lakes, NJ), and DCFH-DA and Hoechst 33258 were purchased from Beyotime Biotechology (Nantong, China).

All other reagents were purchased from Sigma-Aldrich (St. Louis, MO, USA) unless otherwise specified.

### Cells and cell cultures

The human breast cancer cell lines MCF-7, MDA-MB-231 and SUM 159, human liver cancer cell line HepG2, human lung cancer cell line A549, human gastric cancer cell lines SGC-7901 and BGC-823, human mammary epithelial cell line MCF-10A, human skin fibroblast cell line HSF, human lung epithelial cell line L132, human gastric mucosa cell line GES-1 and African green monkey kidney cell line COS-7 were purchased from Life Science Research Institute, Chinese Academy of Sciences, Shanghai. MCF-7, MCF-10A, HepG2, HSF GES-1 and COS-7 cells were cultured in Dulbecco's modified Eagle's medium (DMEM; high glucose, Hyclone, Waltham, MA, USA) supplemented with 10% (v/v) heat-inactivated fetal bovine serum (FBS, Invitrogen, Waltham, MA, USA), 100 μg/ml streptomycin sulfate and 100 U/ml penicillin solution (Gibco, Waltham, MA USA). SGC-7901 and BGC-823 cells were cultured in RPMI 1640 medium (Hyclone) with the same supplementation. A549 and L132 cells were cultured in F12/DMEM medium (Hyclone) with the same supplementation. SUM 159 cells were cultured in F12/DMEM medium (Hyclone, no phenol red) with the same supplementation. All the above cell lines were grown at 37°C in humidified conditions of 95% air and 5% CO_2_. MDA-MB-231 cells were cultured in L-15 medium (Hyclone) with the same supplementation at 37°C in humidified conditions of 100% air.

### Cell viability analysis

Cell viability under GBK treatment was assessed using a Cell Counting Kit-8 (CCK-8) according to the manufacturer's instruction (Beyotime Biotechology). Briefly, cells were seeded at 5 × 10^3^/100 μl per well in 96-well culture plates and incubated in complete medium. After 24 h, culture medium was replaced with fresh medium containing different concentrations of GBK for another 48 h, or cells were treated with fresh medium containing specific concentrations of GBK for 0, 1 d, 3 d or 5 d. At the indicated time points, 10 μl of CCK-8 was added to each well and cells were incubated at 37°C for 4 h. Finally, the absorbance was measured for each well at a wavelength of 450 nm using the Microplate Autoreader (Bio-Tek Instruments Inc., Winooski, VT, USA). Independent experiments were repeated in triplicate.

### Colony formation assay

Cells were seeded in 6-well culture plates in triplicate at a density of 1 × 10^3^/well in 2 ml complete medium. After 24 h, cultures were replaced with fresh medium containing no GBK as control or the same medium containing different concentrations of GBK. The plates were incubated at 37°C with 5% CO_2_ in a humidified incubator for 14 days and media were replaced every third day. The colonies were fixed with 4% paraformaldehyde, stained in 0.5% crystal violet solution for 15 min at room temperature, and washed with distilled water to remove excess dye. The colony numbers were counted under an Axio Imager A2 microscope (Zeiss, Dresden, Germany). Independent experiments were repeated in triplicate.

### Flow cytometry analysis of cell cycle

Flow cytometric analysis was performed to evaluate the effects of GBK on cell cycle progression. In brief, cells were seeded in 6-well culture plates (2 × 10^5^ cells/well) in complete medium. After 24 h, cells were treated with different concentrations of GBK in fresh medium for another 48 h; for time-dependent experiments, cells were treated with specific concentrations of GBK in fresh medium for 12, 24 or 48 h. After treatments, cells were harvested by trypsinization, washed twice with ice-cold PBS, and then fixed overnight at −20°C in 70% ethanol. Cells were washed with PBS and incubated with 50 μg/mL propidium iodide and 50 μg/mL RNase A in PBS on ice for 30 min in the dark. Flow cytometry was performed using a FACSCalibur system (Becton Dickinson, San Jose, CA, USA) with CELLQuest software (Version 3.3, Becton Dickinson) and the cell cycle distributions were calculated by ModFit LT software (Version 3.0, Verity Software House, Topsham, ME, USA). Independent experiments were repeated in triplicate.

### Microarray analysis

Total RNAs were isolated from cells using a Trizol reagent kit (Transgen) according to the manufacturer's instructions. The cDNA was synthesized using TransScript One-Step gDNA Removal and cDNA Synthesis SuperMix (Transgen) following the manufacturer's instructions.

The cDNA fragmentation, labeling, and hybridization of labeled cDNAs from cells with Affymetrix GeneChip^®^ PrimeView™ Human Gene Expression Array and scanning were performed as described in the Affymetrix User Guide (http://www.affymetrix.com). The array includes approximately 530,000 probes covering more than 36,000 transcripts and variants, which represent more than 20,000 genes mapped through RefSeq or via UniGene annotation. Data from three independent experiments were combined to identify candidate genes upregulated or downregulated in response to GBK treatment in MCF-7 and SGC-7901 cell lines (GEO access number GSE87440). Fold change filters required upregulated genes to be present at levels at least 2-fold of controls and for downregulated genes to be 0.5-fold lower than that of controls.

### Relative quantitative real-time PCR

RNA isolated from each sample was from the same pool used for microarray analysis. The cDNA was synthesized using TransScript^®^ One-Step gDNA Removal and cDNA Synthesis SuperMix (Transgen) following the manufacturer's instructions. Briefly, 500 ng of total RNA from cells with or without GBK treatment was used as template in a 20 μl reaction system. The cDNA expression levels of selected candidate genes were measured by RT-qPCR using the primer pairs listed in Supplementary Table 4. Primers for candidate genes and reference gene were designed using the Primer Premier 5.0, with calculated Tm values of 55–62°C and amplification products no longer than 200 bp. cDNA (2 μl) was used as template in a 25 μl reaction mixture including 10 μmol/L of each primer. SYBR^®^*Premix ExTaq*™ II (TliRNaseH Plus) (TaKaRa, Dalian, Japan) was used for the RT-qPCR assay, and the reactions were performed in an Opticon 3 Real-Time PCR System (Bio-Rad, Hercules, California, USA) using the default cycling conditions (an initial incubation at 95°C for 30 s, and 45 cycles of 95°C for 5 s, 60°C for 20 s, and 72°C plate read). Melting curves were run immediately after the last cycle, with the temperature ramping from 65 to 95°C; read every 1.0°C; and held for 10 s at 4°C to exclude any influence of primer dimers. Cycle numbers at which the fluorescence passed the cycle threshold (Ct) were analyzed, and the 2^(-ΔΔCt)^-method was used to calculate the relative expression levels. For each trial, nine independent experiments were performed as three biological replicates and three technical replicates, and all expression levels were normalized to reference gene β-actin.

### Flow cytometry analysis of apoptosis

Cells were seeded in 6-well plates at a density of 2 × 10^5^ cells/well. After 48 h of GBK treatment at 0, 145, 290, and 580 μg/ml, cells were collected. The cells were then washed twice with cold PBS, and 1 × 10^6^ cells were resuspended in 300 μl of 1× binding buffer. Cell suspension (300 μl) was transferred to a 5 ml culture tube and incubated with 5 μl of Annexin V antibody together with 5 μl of PI (Becton Dickinson). Next, 200 μl of 1× binding buffer was added to each tube and the cells were analyzed by flow cytometry within 1 h. Independent experiments were repeated in triplicate.

### Hoechst 33258 staining

At 48 h after GBK (290 or 580 μg/ml) treatment, cells were fixed, washed twice with PBS, and stained with Hoechst 33258 staining solution according to the manufacturer's instructions. The cells were observed and imaged using a confocal microscope LSM 710 (Zeiss) with 60× amplification.

### Caspase activity assays

Caspase-6/9 activity in cell lysates was determined using Caspase 6 or Caspase 9 activity kit (Beyotime Biotechology) according to the manufacturer's protocol. The caspase-6/9 activity was normalized by the protein concentration of the corresponding cell lysate and expressed as percentage of treated cells to that of control. Independent experiments were repeated in triplicate.

### Determination of cellular levels of ROS

Cellular ROS contents were measured by flow cytometry. In brief, cells were seeded in 6-well culture plates (2 × 10^5^ cells/well) in complete medium. After 24 h, cells were treated with GBK (290 μg/ml) in fresh medium for 0, 0.5, 1, 3, or 6 h, or cells were treated with different concentrations of GBK in fresh medium for 0.5 h. Cells were stained with 10 μM DCFH-DA (Beyotime Biotechology) at 37°C for 30 min; cells were then collected and the fluorescence was analyzed using a FACSCalibur flow cytometer (Becton Dickinson). In some experiments, cells were pretreated with 5 mM N-Acetyl-L-cysteine (NAC) (MilliporeSigma) for 2 h prior to GBK exposure and analysis of ROS generation and cell apoptosis.

### Xenograft tumors in NOD/SCID mice

Four-week-old female NOD/SCID mice, obtained from Beijing Vital River Experimental Animals Technology (Beijing, China), were used in all experiments. One week prior to MCF-7 cell implantation, a small incision was made on the back of NOD/SCID mice and a beta-estradiol pellet was embedded. To establish tumors in mice, early passage MCF-7 cells were harvested and resuspended in 1:1 mixture of Matrigel (Becton Dickinson) and PBS. Cells (1 × 10^7^ cells in 100 μl) were then injected subcutaneously into the right and left flank regions of each mouse. After 21 days, the tumors reached a mean diameter of 8 mm in all recipients. Tumor-bearing mice were randomly divided into control and treated groups (4 mice as control, and 8 mice as the experiment group). Based on preliminary experiments, GBK was intraperitoneally administered at 10 mg/kg body weight per day for 3 weeks in the GBK-treatment group, and the control group received vehicle only. Body weight and diet consumption were recorded twice weekly throughout the study. After the initiation of GBK administration, the tumor size was measured with an external caliper twice a week and tumor volume was calculated using the following formula: tumor volume V=ab^2^/2, in which “a” represents the long axis and “b” represents the short axis of the tumor. At the end of experiment, all the animals were sacrificed and the tumors were excised, weighed, fixed in 4% paraformaldehyde and embedded in paraffin for additional analyses.

### Immunohistochemistry

All tumor tissues were fixed with 4% paraformaldehyde (pH 7.4) and embedded in paraffin. Paraffin-embedded tissue sections (5-μm thick) were processed and immunohistochemistry was performed. In brief, sections were deparaffinized in xylene and rehydrated in ethanol. Sections were rehydrated and endogenous enzyme was blocked for 10 min, followed by 3 min of heat-induced antigen retrieval. Sections were then incubated with primary antibodies (cyclin E, MCM2, VEGF, CDC6, or BCL2) at 4°C overnight. On the next day, after 45 min of incubation with HRP Conjugate Goat Anti-Rabbit IgG (H+L) antibody reagent at 37°C, the signals were detected by adding substrate hydrogen peroxide using diaminobenzidine as a chromogen followed by hematoxylin counterstaining. All sections were examined under a light microscope (Zeiss).

### Statistical analysis

Data are presented as the mean ± SE from three independent experiments, and all statistical analyses were performed using a Student's t test with SPSS 20.0 software (SPSS Inc., Chicago, IL, USA). Significant differences were accepted when *P*<0.05.

## SUPPLEMENTARY MATERIALS FIGURES AND TABLES





## References

[1] Torre LA, Bray F, Siegel RL, Ferlay J, Lortet-Tieulent J, Jemal A (2015). Global cancer statistics, 2012. CA Cancer J Clin.

[2] Fulford LG, Easton DF, Reis-Filho JS, Sofronis A, Gillett CE, Lakhani SR, Hanby A (2006). Specific morphological features predictive for the basal phenotype in grade 3 invasive ductal carcinoma of breast. Histopathology.

[3] Mislang AR, Biganzoli L (2015). Adjuvant Systemic Therapy in. Older Breast Cancer Women: Can We Optimize the Level of Care? Cancers.

[4] Leone J, Leone BA, Leone JP (2016). Adjuvant systemic therapy in older women with breast cancer. Breast cancer.

[5] Balasubramanian BA, Gandhi SK, Demissie K, August DA, Kohler B, Osinubi OY, Rhoads GG (2007). Use of adjuvant systemic therapy for early breast cancer among women 65 years of age and older. Cancer control.

[6] Pennisi A, Kieber-Emmons T, Makhoul I, Hutchins L (2016). Relevance of Pathological Complete Response after Neoadjuvant Therapy for Breast Cancer. Breast cancer.

[7] Dai X, Xiang L, Li T, Bai Z (2016). Cancer Hallmarks, Biomarkers and Breast Cancer Molecular Subtypes. Journal of Cancer.

[8] Gu G, Dustin D, Fuqua SA (2016). Targeted therapy for breast cancer and molecular mechanisms of resistance to treatment. Current opinion in pharmacology.

[9] Saini KS, Azim HA, Metzger-Filho O, Loi S, Sotiriou C, de Azambuja E, Piccart M (2011). Beyond trastuzumab: new treatment options for HER2-positive breast cancer. Breast.

[10] Hanahan D, Weinberg RA (2011). Hallmarks of cancer: the next generation. Cell.

[11] Hainaut P, Plymoth A (2013). Targeting the hallmarks of cancer: towards a rational approach to next-generation cancer therapy. Current opinion in oncology.

[12] Chen X, Lu P, Wu Y, Wang DD, Zhou S, Yang SJ, Shen HY, Zhang XH, Zhao JH, Tang JH (2016). MiRNAs-mediated cisplatin resistance in breast cancer. Tumour biology.

[13] Damiani RM, Moura DJ, Viau CM, Caceres RA, Henriques JA, Saffi J (2016). Pathways of cardiac toxicity: comparison between chemotherapeutic drugs doxorubicin and mitoxantrone. Archives of toxicology.

[14] Li J, Ren J, Sun W (2017). Systematic review of ixabepilone for treating metastatic breast cancer. Breast cancer.

[15] Jin Z, Borjihan G, Zhao R, Sun Z, Hammond GB, Uryu T (2009). Antihyperlipidemic compounds from the fruit of Piper longum L. Phytotherapy research.

[16] Sarnaizul E, Borjihan G, Baigude H, Aona, Menghe, Narisu, Zhaorigetu (2013). LC analysis and pharmacokinetic study of synthetic piperlonguminine in rat plasma after oral administration. Biomedical chromatography.

[17] Raj L, Ide T, Gurkar AU, Foley M, Schenone M, Li X, Tolliday NJ, Golub TR, Carr SA, Shamji AF, Stern AM, Mandinova A, Schreiber SL, Lee SW (2011). Selective killing of cancer cells by a small molecule targeting the stress response to ROS. Nature.

[18] Bezerra DP, Militao GC, de Castro FO, Pessoa C, de Moraes MO, Silveira ER, Lima MA, Elmiro FJ, Costa-Lotufo LV (2007). Piplartine induces inhibition of leukemia cell proliferation triggering both apoptosis and necrosis pathways. Toxicology in vitro.

[19] Johnson GL, Lapadat R (2002). Mitogen-activated protein kinase pathways mediated by ERK, JNK, and p38 protein kinases. Science.

[20] Chen W, Liu L, Luo Y, Odaka Y, Awate S, Zhou H, Shen T, Zheng S, Lu Y, Huang S (2012). Cryptotanshinone activates p38/JNK and inhibits Erk1/2 leading to caspase-independent cell death in tumor cells. Cancer prevention research.

[21] Zou P, Xia Y, Chen T, Zhang J, Wang Z, Chen W, Chen M, Kanchana K, Yang S, Liang G (2016). Selective killing of gastric cancer cells by a small molecule targeting ROS-mediated ER stress activation. Molecular carcinogenesis.

[22] Lei M (2005). The MCM complex: its role in DNA replication and implications for cancer therapy. Current cancer drug targets.

[23] Santamaria D, Ortega S (2006). Cyclins and CDKS in development and cancer: lessons from genetically modified mice. Frontiers in bioscience.

[24] Sandal T (2002). Molecular aspects of the mammalian cell cycle and cancer. The oncologist.

[25] Zhu W, Depamphilis ML (2009). Selective killing of cancer cells by suppression of geminin activity. Cancer research.

[26] Lim W, Park S, Bazer FW, Song G (2016). Naringenin-Induced Apoptotic Cell Death in Prostate Cancer Cells Is Mediated via the PI3K/AKT and MAPK Signaling Pathways. Journal of cellular biochemistry.

[27] Chen JC, Hsieh MJ, Chen CJ, Lin JT, Lo YS, Chuang YC, Chien SY, Chen MK (2016). Polyphyllin G induce apoptosis and autophagy in human nasopharyngeal cancer cells by modulation of AKT and mitogen-activated protein kinase pathways in vitro and in vivo. Oncotarget.

[28] He JD, Wang Z, Li SP, Xu YJ, Yu Y, Ding YJ, Yu WL, Zhang RX, Zhang HM, Du HY (2016). Vitexin suppresses autophagy to induce apoptosis in hepatocellular carcinoma via activation of the JNK signaling pathway. Oncotarget.

[29] Slieker RC, Roost MS, van Iperen L, Suchiman HE, Tobi EW, Carlotti F, de Koning EJ, Slagboom PE, Heijmans BT, Chuva de Sousa Lopes SM (2015). DNA Methylation Landscapes of Human Fetal Development. PLoS genetics.

[30] Suzuki MM, Bird A (2008). DNA methylation landscapes: provocative insights from epigenomics. Nature reviews Genetics.

[31] Nazarian R, Jazirehi AR (2014). Epigenomics and targeted therapy in cancer. Epigenomics.

[32] Esteller M (2007). Cancer epigenomics: DNA methylomes and histone-modification maps. Nature reviews Genetics.

[33] Zhang L, Yang W, Zhu X, Wei C (2016). p53 inhibits the expression of p125 and the methylation of POLD1 gene promoter by downregulating the Sp1-induced DNMT1 activities in breast cancer. OncoTargets and therapy.

[34] Radpour R, Barekati Z, Haghighi MM, Kohler C, Asadollahi R, Torbati PM, Holzgreve W, Zhong XY (2010). Correlation of telomere length shortening with promoter methylation profile of p16/Rb and p53/p21 pathways in breast cancer. Modern pathology.

[35] Cai FF, Chen S, Wang MH, Lin XY, Zhang L, Zhang JX, Wang LX, Yang J, Ding JH, Pan X, Shao ZM, Biskup E (2016). Pyrosequencing quantified methylation level of BRCA1 promoter as prognostic factor for survival in breast cancer patient. Oncotarget.

[36] Zhang L, Long X (2015). Association of BRCA1 promoter methylation with sporadic breast cancers: Evidence from 40 studies. Scientific reports.

[37] Hennessy BT, Gonzalez-Angulo AM, Stemke-Hale K, Gilcrease MZ, Krishnamurthy S, Lee JS, Fridlyand J, Sahin A, Agarwal R, Joy C, Liu W, Stivers D, Baggerly K (2009). Characterization of a naturally occurring breast cancer subset enriched in epithelial-to-mesenchymal transition and stem cell characteristics. Cancer research.

[38] Cedar H, Bergman Y (2009). Linking DNA methylation and histone modification: patterns and paradigms. Nature reviews Genetics.

[39] Dong C, Wu Y, Wang Y, Wang C, Kang T, Rychahou PG, Chi YI, Evers BM, Zhou BP (2013). Interaction with Suv39H1 is critical for Snail-mediated E-cadherin repression in breast cancer. Oncogene.

[40] Cragg GM, Grothaus PG, Newman DJ (2009). Impact of natural products on developing new anti-cancer agents. Chemical reviews.

[41] Zhang F, Xu L, Qu X, Zhao M, Jin B, Kang J, Liu Y, Hu X (2011). Synergistic antitumor effect of beta-elemene and etoposide is mediated via induction of cell apoptosis and cell cycle arrest in non-small cell lung carcinoma cells. Molecular medicine reports.

[42] Dinic J, Podolski-Renic A, Stankovic T, Bankovic J, Pesic M (2015). New Approaches With Natural Product Drugs for Overcoming Multidrug Resistance in Cancer. Current pharmaceutical design.

[43] Bishayee A, Sethi G (2016). Bioactive natural products in cancer prevention and therapy: Progress and promise. Seminars in cancer biology.

[44] Sebti SM (2005). Protein farnesylation: implications for normal physiology, malignant transformation, and cancer therapy. Cancer cell.

[45] Hindler K, Cleeland CS, Rivera E, Collard CD (2006). The role of statins in cancer therapy. The oncologist.

[46] Pisanti S, Picardi P, Ciaglia E, D'Alessandro A, Bifulco M (2014). Novel prospects of statins as therapeutic agents in cancer. Pharmacological research.

[47] Gazzerro P, Proto MC, Gangemi G, Malfitano AM, Ciaglia E, Pisanti S, Santoro A, Laezza C, Bifulco M (2012). Pharmacological actions of statins: a critical appraisal in the management of cancer. Pharmacological reviews.

[48] Obiorah IE, Fan P, Sengupta S, Jordan VC (2014). Selective estrogen-induced apoptosis in breast cancer. Steroids.

[49] Erdenebaatar S, Gereltu B, Sun Z (2013). The preparation and antihyperlipidaemic assay of piperlonguminine in vivo. Phytochemistry letters.

