# Supplementary material for: The synthetic antihyperlipidemic drug potassium piperate selectively kills breast cancer cells through inhibiting G1-S-phase transition and inducing apoptosis
Source: Oncotarget. 2017 Apr 12;8(29):47250–68. doi: 10.18632/oncotarget.16872 (PMC5564562; doi:10.18632/oncotarget.16872)
Supplement: Supplementary file 1 [file oncotarget-08-47250-s001.pdf]

The synthetic antihyperlipidemic drug potassium piperate selectively kills breast cancer cells through inhibiting G1-S-phase transition and inducing apoptosis

SUPPLEMENTARY MATERIALS

SUPPLEMENTARY FIGURES AND TABLES

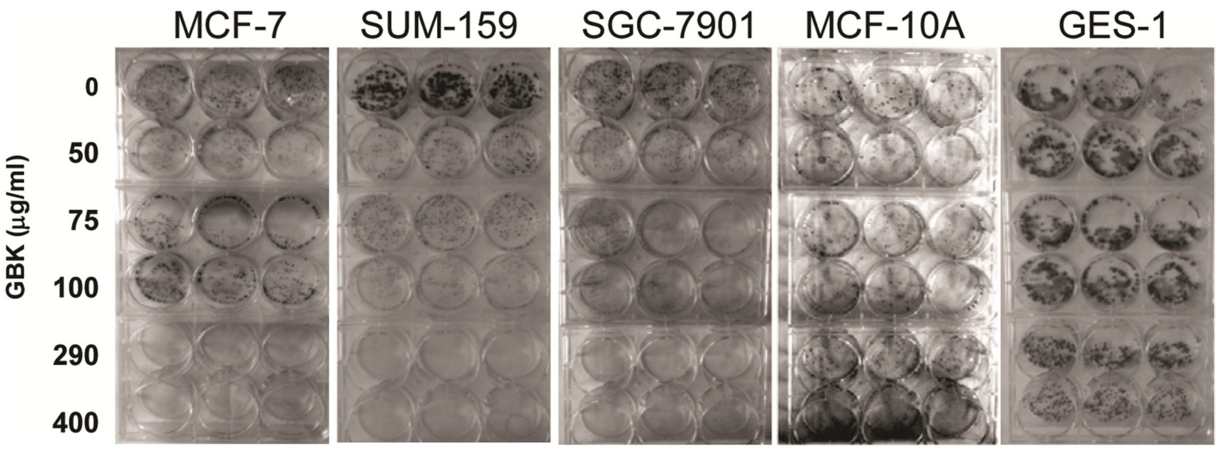

Supplementary Figure 1: Selective killing of tumor cells by GBK in a dose-dependent manner. Normal and tumor cells were treated with GBK at 0-400 µg/ml concentrations for 14 days, and live cells were stained by crystal violet. ddH_2O was used as control solvent. GBK exhibited cytotoxicity only in tumor cells (MCF-7, SUM159 and SGC-7901) and not in normal human breast epithelial cells (MCF-10A) and human gastric mucosa cells (GES-1) at less than 290 µg/ml concentration. At higher concentration of GBK (>400 µg/ml) slight cytotoxicity was observed in normal human breast epithelial cells.

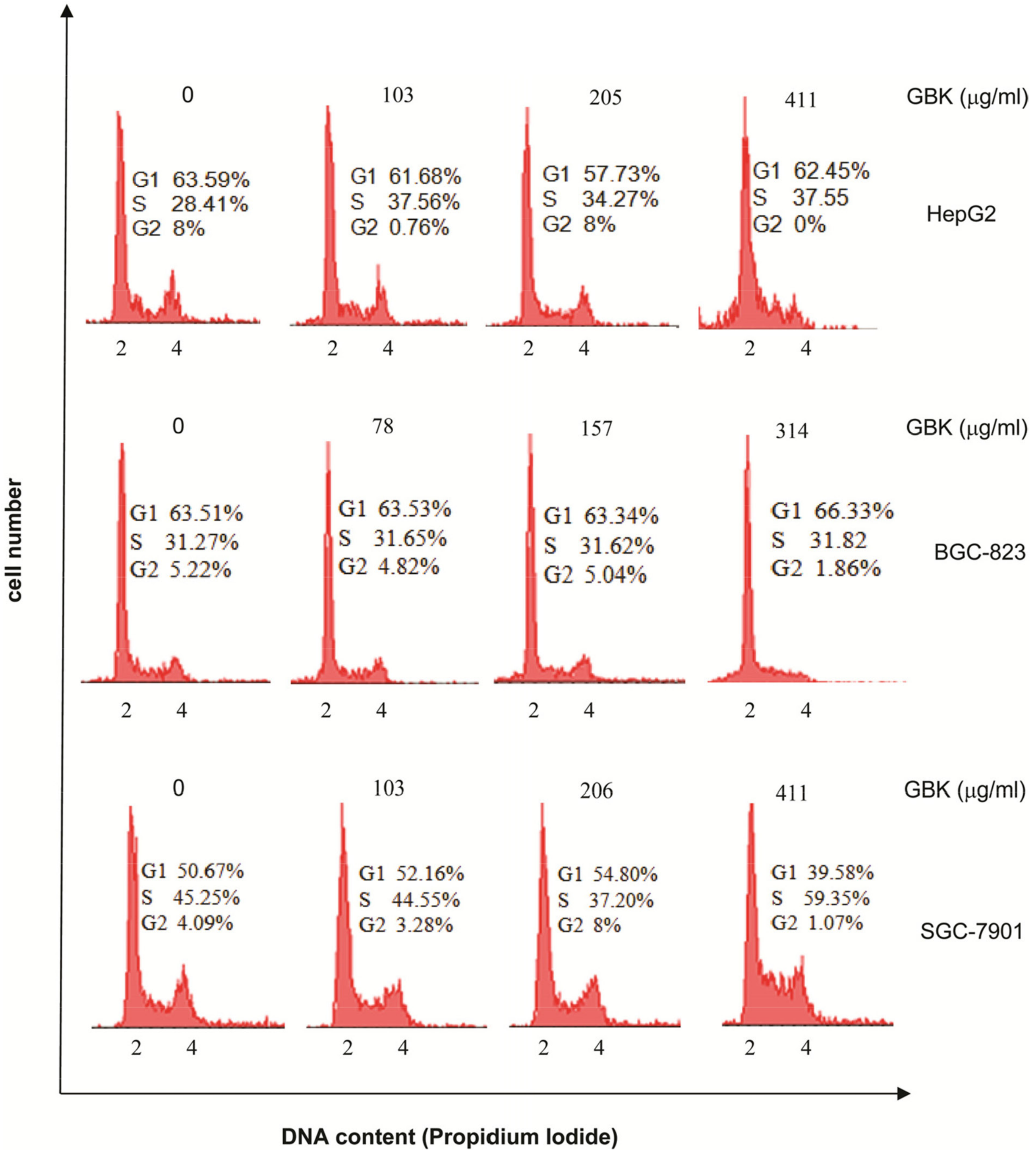

Supplementary Figure 2: GBK induces G1/S arrest in breast cancer cell line MCF-7 *in vitro* but not in other cancer cell lines. Three different cancer cell lines (HepG2, BGC-823 and SGC-7901) were treated with different concentrations of GBK for 48 h respectively according to their different IC_{50} values, and cell cycle distributions were analyzed by flow cytometry. ddH_2O was used as control.

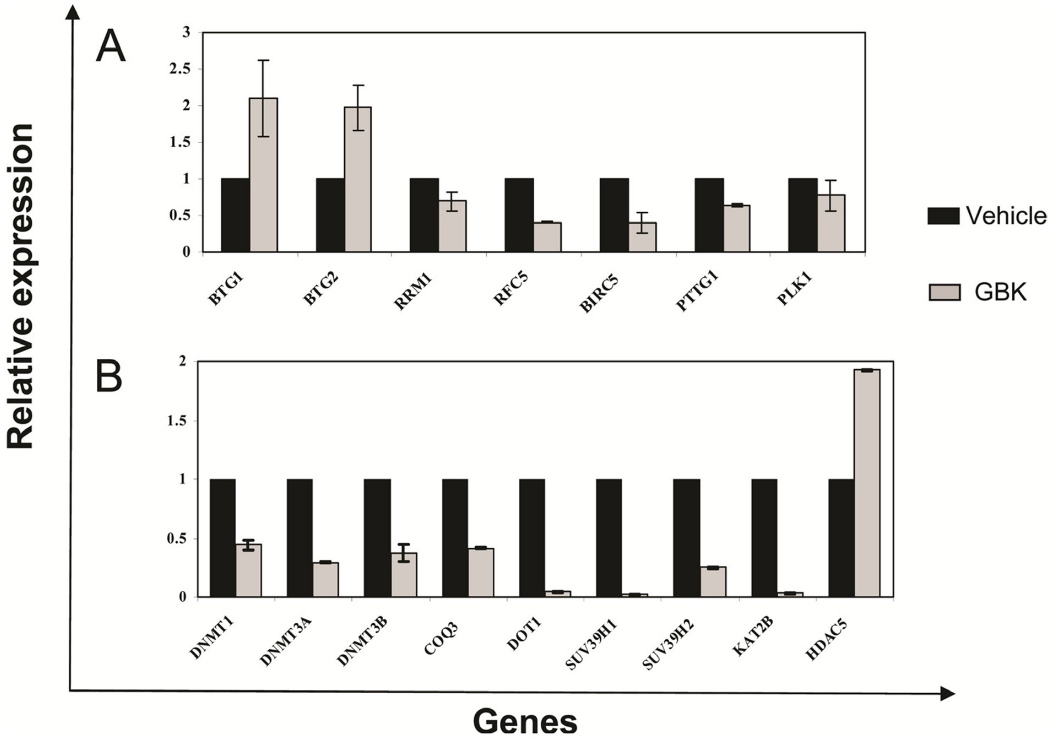

Supplementary Figure 3: Differential expression of genes in MCF-7 cells treated with GBK. Differential expression of genes in MCF-7 cells after treatment with GBK were analyzed by RT-qPCR. **(A)** Genes related to cancer progression were selected. BTG1/2 are members of a family of antiproliferative genes, RRMI is ribonucleotide reductase M1, RFC5 is the replication factor C which is required for DNA replication, BIRC5 is a member of the inhibitor of apoptosis (IAP) gene family, PTTG1 is pituitary tumor-transforming 1which is highly expressed in various tumors, and PLK1 is Polo-like kinase 1 which is also highly expressed in various tumors. **(B)** Genes related to DNA methylation, histone methylation and acetylation were selected. DNMT1, DNMT3A, DNMT3B and COQ3 are DNA methyltransferase. DOT1, SUV39H1 and SUV39H2 are histone methyltransferase. KAT2B is K (lysine) acetyltransferase 2B and HDAC5 is histone deacetylase 5.

Supplementary Table 1: IC_{50} of human cancer cells and normal cells treated with GBK

Cell lines	Remark	IC_{50} (μg/ml)
MCF-7	Human breast cancer cells	291.5
MDA-MB-231	Human breast cancer cells	295
SUM-159	Human breast cancer cells	250
HepG2	Human liver cancer cells	205.4
A549	Human lung cancer cells	191.5
SGC-7901	Human gastric cancer cells	205.6
BGC-823	Human gastric cancer cells	156.8
MCF-10A	Human mammary epithelial cells	——
HSF	Human skin fibroblast cells	——
GES-1	Human gastric mucosa cells	——
L132	Human lung epithelial cells	——
COS-7	African green monkey kidney cells	——

The results suggest that GBK has cytotoxicity on different human cancer cell lines, but not on normal human cells. The IC_{50} of human breast cancer cells treated with GBK ranges from 250 μg/ml to 295 μg/ml.

Supplementary Table 2: Differential expression of genes in MCF-7 cells treated with GBK

See Supplementary File 1

Supplementary Table 3: Differential expression of genes in SGC-7901 cells treated with GBK

See Supplementary File 1

Supplementary Table 4: Primers used in RT-qPCR to validate microarray data

See Supplementary File 1

Supplementary Table 5: Expression profiling of GBK-responsive gene transcripts involved in cell apoptosis selected from Affymetrix GeneChip® PrimeView™ Human Gene Expression Array

Gene ID	Expression fold	Description	Gene symbol	Signaling pathway
11717364_a_at	0.4714	BCL2-like 12	Bcl-2L12	Apoptosis
11754110_x_at	0.4356	baculoviral IAP repeat-containing 5	BIRC5	Apoptosis
11737440_a_at	2.2025	BCL2 modifying factor	BMF	Apoptosis
11759444_x_at	1.1636	BCL2-associated X protein	BAX	Apoptosis
11731870_a_at	1.1634	NADPH oxidase activator 1	NOXA	Apoptosis
11757047_x_at	1.4219	activating transcription factor 4	ATF4	Smooth muscle contraction

GBK treatment and global transcription microarray analysis for MCF-7 cells were as described in methods and text. The relative expressions of genes involved either in the apoptotic or pro-survival responses were calculated by finding ratio of the values of GBK treatment group to that of vehicle treatment group. The expression fold < 0.5 indicates down-regulated expressions. The expression fold > 1 shows up-regulated expression. The gene ID indicates the Entry number of NCBI.
