# Supplementary material for: The synthetic antihyperlipidemic drug potassium piperate selectively kills breast cancer cells through inhibiting G1-S-phase transition and inducing apoptosis
Source: Oncotarget. 2017 Apr 12;8(29):47250–68. doi: 10.18632/oncotarget.16872 (PMC5564562; doi:10.18632/oncotarget.16872)
Supplement: Supplementary file 2 [file oncotarget-08-47250-s002.doc]

**Table S2: Differential expression of genes in MCF-7 cells treated with GBK**

| **Gene ID** | **Expression fold** | **Description** | **Gene symbol** | **Signaling pathway** |
| --- | --- | --- | --- | --- |
| 11750410 | 8.0894 | UDP glucuronosyltransferase 1 family, polypeptide A1 | UGT1A1 | Irinotecan pathway |
| 11715435 | 5.9874 | TIMP metallopeptidase inhibitor 3 | TIMP3 | Matrix Metalloproteinase |
| 11725363 | 2.0147 | SMAD family member 4 | SMAD4 | Cell cycle |
| 11718579 | 2.1571 | glutathione S-transferase mu 3 | GSTM3 | Circadian Exercise |
| 11733024 | 2.0547 | B-cell translocation gene 1 | BTG1 | Circadian Exercise |
| 11756666 | 0.3662 | Minichromosome maintenance complex component 2 | MCM2 | Cell cycle |
| 11749478 | 0.4409 | minichromosome maintenance complex component 3 | MCM3 | Cell cycle |
| 11736670 | 0.3836 | minichromosome maintenance complex component 4 | MCM4 | Cell cycle |
| 11732997 | 0.3799 | minichromosome maintenance complex component 6 | MCM6 | Cell cycle |
| 11752905 | 0.3980 | minichromosome maintenance complex component 7 | MCM7 | Cell cycle |
| 11734971 | 0.4263 | minichromosome maintenance complex component 8 | MCM8 | Cell cycle |
| 11736367 | 0.2665 | minichromosome maintenance complex component 10 | MCM10 | Cell cycle |
| 11723713 | 0 .4598 | E2F transcription factor 1 | E2F1 | Cell cycle |
| 11724079 | 0.2665 | E2F transcription factor 2 | E2F2 | Cell cycle |
| 11730029 | 0 .2189 | E2F transcription factor 7 | E2F7 | Cell cycle |
| 11728958 | 0.3260 | E2F transcription factor 8 | E2F8 | Cell cycle |
| 11743064 | 0.3208 | cell division cycle 6 homolog | CDC6 | Cell cycle |
| 11745049 | 0 .3416 | cell division cycle 45 homolog | CDC45 | Cell cycle |
| 11718488 | 0 .4170 | origin recognition complex, subunit 3 | ORC3 | Cell cycle |
| 11727837 | 0.3522 | origin recognition complex, subunit 6 | ORC6 | Cell cycle |
| 11716103 | 0.4044 | proliferating cell nuclear antigen | PCNA | DNA replication Reactome |
| 11721666 | 0.4354 | chromatin licensing and DNA replication factor 1 | CDT1 | DNA replication Reactome |
| 11754937 | 0.3905 | geminin, DNA replication inhibitor | GMNN | DNA replication Reactome |
| 11733102 | 0.4839 | polymerase (DNA-directed), delta 3, | POLD3 | DNA replication Reactome |
| 11744519 | 0.1949 | polymerase (DNA directed), epsilon 2 | POLE2 | DNA replication Reactome |
| 11739763 | 0.4260 | GINS complex subunit 3 | GINS3 | DNA replication Reactome |
| 11722571 | 0.2823 | cyclin A2 | CCNA2 | Cell cycle |
| 11723939 | 0.3472 | cyclin B1 | CCNB1 | Cell cycle |
| 11716793 | 0.3207 | Cyclin B2 | CCNB2 | Cell cycle |
| 11728300 | 0.2622 | cyclin E2 | CCNE2 | Cell cycle |
| 11759013 | 0.4746 | CHK1 checkpoint homolog | CHEK1 | Cell cycle |
| 11721893 | 0.4379 | CHK2 checkpoint homolog | CHEK2 | Cell cycle |
| 11727536 | 0.4454 | cyclin F | CCNF | Cell cycle |
| 11753788 | 0 .3453 | cyclin-dependent kinase inhibitor 3 | CDKN3 | Cell cycle |
| 11736405 | 0.4913 | DNA(cytosine-5)-methyltransferase1 | DNMT1 | S1P Signaling |
| 11727791 | 0.4852 | dihydrolipoamide S-acetyltransferase | DLAT | Glycolysis and Gluconeogenesis |
| 11757538 | 0.4784 | glutamic-oxaloacetic transaminase 1 | GOT1 | Glycolysis and Gluconeogenesis |
| 11743603 | 0.4719 | ribonucleotide reductase M1 | RRM1 | Nucleotide Metabolism |
| 11744232 | 0.4716 | replication factor C (activator 1) 5 | RFC5 | DNA replication Reactome |
| 11735662 | 0.4703 | purinergic receptor P2Y | P2RY2 | Nucleotide GPCRs |
| 11723583 | 0.4415 | Rac GTPase activating protein 1 | RACGAP1 | S1P Signaling |
| 11754110 | 0.4356 | baculoviral IAP repeat-containing 5 | BIRC5 | Apoptosis |
| 11717521 | 0.4216 | pituitary tumor-transforming 1 | PTTG1 | Cell cycle |
| 11716726 | 0.4427 | polo-like kinase 1 | PLK1 | Cell cycle |

GBK treatment and global transcription microarray analysis for MCF-7 cells were as described in methods and text. The relative expressions of genes were calculated by finding ratio of the values of GBK treatment group to that of vehicle treatment group. The expression fold < 0.5 indicates down-regulated expressions. The expression fold > 2 shows up-regulated expression. The gene ID indicates the entry number of NCBI.

**Table S3: Differential expression of genes in** **SGC-7901 cells treated with GBK**

| **Gene ID** | **Expression fold** | **Description** | **Gene symbol** | **Signaling pathway** |
| --- | --- | --- | --- | --- |
| 11716974 | 3.2421 | "pyruvate dehydrogenase kinase, isozyme 4" | PDK4 | Krebs TCA Cycle |
| 11752940 | 2.5401 | early growth response 1 | EGR1 | Ovarian Infertility Genes |
| 11719488 | 2.1944 | endothelin receptor type A | EDNRA | GPCRDB Class A Rhodopsin like |
| 11719556 | 2.1566 | "pyruvate dehydrogenase kinase, isozyme 2" | PDK2 | Krebs TCA Cycle |
| 11720109 | 2.0669 | cAMP responsive element binding protein 3-like 4 | CREB3L4 | G1 to S cell cycle Reactome |
| 11721668 | 2.2982 | chromatin licensing and DNA replication factor 1 | CDT1 | DNA replication Reactome |
| 11724032 | 2.3041 | follistatin | FST | TGF Beta Signaling Pathway |
| 11724478 | 2.1261 | FOS-like antigen 1 | FOSL1 | Wnt signaling |
| 11747474 | 2.0686 | "nuclear receptor subfamily 4, group A, member 2" | NR4A2 | Nuclear Receptors |
| 11726337 | 5.2782 | arachidonate 5-lipoxygenase | ALOX5 | Eicosanoid Synthesis |
| 11726624 | 2.422 | cyclin G2 | CCNG2 | G1 to S cell cycle Reactome |
| 11728189 | 2.1681 | chemokine (C-X-C motif) receptor 4 | CXCR4 | GPCRDB Class A Rhodopsin like |
| 11728209 | 2.0683 | "protein phosphatase 2, regulatory subunit B, gamma" | PPP2R2C | Glycogen Metabolism |
| 11729365 | 2.5234 | "protein kinase, AMP-activated, alpha 2 catalytic subunit" | PRKAA2 | Fatty Acid Synthesis |
| 11732111 | 3.0209 | "tumor necrosis factor receptor superfamily, member 21" | TNFRSF21 | Apoptosis |
| 11739503 | 2.2752 | "ATP-binding cassette, sub-family A (ABC1), member 1" | ABCA1 | Statin Pathway PharmGKB |
| 11740638 | 2.3753 | noggin | NOG | TGF Beta Signaling Pathway |
| 11742775 | 3.0109 | G protein-coupled receptor 161 | GPR161 | GPCRDB Class A Rhodopsin like2 |
| 11743617 | 2.5134 | "integrin, alpha 2 (CD49B, alpha 2 subunit of VLA-2 receptor)" | ITGA2 | Integrin mediated cell adhesion |
| 11743747 | 2.1725 | "phosphorylase kinase, alpha 1 (muscle)" | PHKA1 | Glycogen Metabolism |
| 11743949 | 2.4974 | frequently rearranged in advanced T-cell lymphomas | FRAT1 | Wnt signaling |
| 11758879 | 2.2143 | "major histocompatibility complex, class I, E" | HLA-E | Proteasome Degradation |
| 11745021 | 2.132 | v-myc myelocytomatosis viral oncogene homolog (avian) | MYC | Apoptosis |
| 11745380 | 3.7145 | "solute carrier family 25, member 27" | SLC25A27 | Electron Transport Chain |
| 11746229 | 4.1925 | ribosomal protein L31 | RPL31 | Ribosomal Proteins |
| 11756651 | 2.4432 | "TAF12 RNA polymerase II, TATA box binding protein (TBP)-associated factor, 20kDa" | TAF12 | RNA transcription Reactome |
| 11757987 | 2.017 | POZ (BTB) and AT hook containing zinc finger 1 | PATZ1 | Translation Factors |
| 11758936 | 2.0367 | heterogeneous nuclear ribonucleoprotein R | HNRNPR | mRNA processing Reactome |
| 11759113 | 2.4722 | ribosomal protein L37 | RPL37 | Ribosomal Proteins |
| 11763584 | 2.0269 | Mitogen-activated protein kinase kinase 5 | MAP2K5 | Integrin mediated cell adhesion |
| 11715273 | 0.4663 | tropomyosin 2 (beta) | TPM2 | Striated muscle contraction |
| 11715467 | 0.3246 | Rho GDP dissociation inhibitor (GDI) beta | ARHGDIB | G13 Signaling Pathway |
| 11716906 | 0.165 | "cadherin 1, type 1, E-cadherin (epithelial)" | CDH1 | Cell cycle |
| 11717473 | 0.4212 | insulin-like growth factor binding protein 1 | IGFBP1 | Smooth muscle contraction |
| 11721135 | 0.3545 | insulin-like growth factor binding protein 3 | IGFBP3 | Smooth muscle contraction |
| 11721924 | 0.2885 | "protein kinase C, beta" | PRKCB | Calcium regulation in cardiac cells |
| 11722992 | 0.3561 | dystrophin | DMD | Striated muscle contraction |
| 11723069 | 0.1207 | "gamma-aminobutyric acid (GABA) B receptor, 1 /// ubiquitin D" | GABBR1 /// UBD | GPCRDB Class C Metabotropic glutamate pheromone |
| 11724013 | 0.4825 | sterol O-acyltransferase 1 | SOAT1 | Statin Pathway PharmGKB |
| 11724904 | 0.4974 | TIMP metallopeptidase inhibitor 4 | TIMP4 | Matrix Metalloproteinases |
| 11725198 | 0.3484 | "interleukin 1, alpha" | IL1A | Hypertrophy model |
| 11726496 | 0.4559 | ryanodine receptor 2 (cardiac) | RYR2 | Calcium regulation in cardiac cells |
| 11727092 | 0.4079 | interleukin 18 (interferon-gamma-inducing factor) | IL18 | Hypertrophy model |
| 11734013 | 0.4607 | bone morphogenetic protein 4 | BMP4 | TGF Beta Signaling Pathway |
| 11734711 | 0.458 | G protein-coupled receptor 37 like 1 | GPR37L1 | GPCRDB Class A Rhodopsin-like |
| 11740489 | 0.4107 | mitogen-activated protein kinase 9 | MAPK9 | TGF Beta Signaling Pathway |
| 11746463 | 0.1749 | "interleukin 6 (interferon, beta 2)" | IL6 | Smooth muscle contraction |
| 11753139 | 0.3842 | annexin A8 /// annexin A8-like 1 /// annexin A8-like 2 | ANXA8 /// ANXA8L1 /// ANXA8L2 | Prostaglandin synthesis regulation |
| 11755665 | 0.4569 | "valyl-tRNA synthetase 2, mitochondrial (putative)" | VARS2 | RNA transcription Reactome |
| 11744031 | 0.1722 | "matrix metallopeptidase 7 (matrilysin, uterine)" | MMP7 | Matrix Metalloproteinases |

GBK treatment and global transcription microarray analysis for SGC-7901 cells were as described in methods and text. The relative expressions of genes were calculated by finding ratio of the values of GBK treatment group to that of vehicle treatment group. The expression fold < 0.5 indicates down-regulated expressions. The expression fold > 2 shows up-regulated expression. The gene ID indicates the entry number of NCBI.

**Table S4: Primers used in RT-qPCR to validate microarray data**

| **Gene name** | **Forward primer sequence** | **Reverse primer sequence** | **Size** |
| --- | --- | --- | --- |
| MCM2 | AGACTTTTGCCCGCTACCTTT | TATGTCACCTGCTCTGCCACTA | 91 |
| MCM3 | ACACACTCCAAAGACGGCAGA | ATGGATTCTGTGAGGCGATTC | 156 |
| MCM4 | GCGGGACAAGGAAGGATTTT | GCAGATGCCGTTGTCACTCAG | 257 |
| MCM6 | TGAGCGGAACTTTTCTGTGC | TTACTTCTAAACTGCGGGGGAT | 225 |
| MCM7 | CCGACCGAGACAATGACCTAC | CTCCACCACATCCACCATTC | 205 |
| MCM8 | TGGCTATGCTCGGCAGTATG | CTGCCTGGTAGTGATTGGTGAG | 127 |
| MCM10 | TTAGCAGAAGCCAAAAAGTTAGC | CCTCAGATTCCAGATAGGCAAGT | 229 |
| CCNA2 | TGTCACCGTTCCTCCTTGG | GGGCATCTTCACGCTCTATTT | 125 |
| CCNB1 | CTGGATAATGGTGAATGGACA | GCCTTGGCTAAATCTTGAACT | 221 |
| CCNB2 | CAACTGAAACCTACTGCTTCTGTC | CAATCTTCGTTATCAATGTCCTCG | 164 |
| CCNE2 | CCTCAGGTTTGGAGTGGGAC | TCAGTGCTCTTCGGTGGTGT | 256 |
| CCNF | CCCCAAGGACTACAGGCAA | CTGTTCTCCCGCTTCCGTTT | 228 |
| CDC6 | ATCCCAGGCACAGGCTACA | AGGCAGGGCTTTTACACGAG | 142 |
| CDC45 | GAACTTTTTTGGGAGGGCG | ACAGGAGGGAAATAAGTGCGTC | 140 |
| CDT1 | TGATGCGTAGGCGTTTTGAG | TCCTGGTGCCATCCTTGAA | 114 |
| ORC3 | GTCTCCTCCTGTTGTCGTTATCTTG | TGAGGAAGCAATCGGTGGA | 162 |
| ORC6 | CCCAGCAAAGGAAATGGAGAA | TTTTGAGCACTGGCAGCATT | 123 |
| GMNN | TCTCAGTATTGGAAGGAAGTGGC | TTCTCCTTTTTCAGGCGGG | 128 |
| GINS3 | ACACTTCAGCCCTGGTAGCC | CTGCCCCTTCTCCCAACACT | 101 |
| PCNA | CAGGGCTCCATCCTCAAGAA | GGTCGCAGCGGTAGGTGTC | 169 |
| POLD3 | AAAAAAAGGGGGAAGCGAGT | GGTTCAAGAGGTGGAGACGG | 191 |
| POLE2 | ATTTCCACCCACTGAGCCC | CCAGCAAACATTATGCGAAGT | 207 |
| E2F1 | CATCAGTACCTGGCCGAGAG | TGGTGGTCAGATTCAGTGAGG | 200 |
| E2F2 | AGAACAACATCCAGTGGGTAGG | GCCTGCTCCGTGTTCATCA | 109 |
| E2F7 | AGGGATGGAGGTAAATTGTTTAACACT | TTTCCCCATCTTCAACTGCAA | 159 |
| E2F8 | CTGATCTGCGAACAGGATATTAAAAC | AAAATGAAAAATCTGGAGTTCCTCC | 186 |
| Cyclin D1 | AAGGAGATTGTGCCATCCATGC | CGGCTCTTCTTCAGGGGCTCC | 181 |
| Cyclin E | GAGCCAGCCTTGGGACAATAA | GCACGTTGAGTTTGGGTAAACC | 103 |
| p53 | CCGCAGTCAGATCCT AGCG | AATCATCCATTGCTTGGGACG | 118 |
| Rb | ATCCGAGGCAACTACAGCCTA | CCTTTCCAACCGTGGGAATAAT | 100 |
| CDK2 | CCCAGATGAGGTGGTGTGG | CCGCTTGTTAGGGTCGTAGTC | 160 |
| BTG1 | ATTGGACAGGCAGCACAGC | TTCATACAGCACACAGATGGAGC | 141 |
| BTG2 | CCTATGAGGTGTCCTACCGC | CACGTAGTTCTTGGAGGGGC | 143 |
| RRM1 | CAGGAAAGGAAGAGCAGCG | CCAGGACACTCATTTGGACACA | 121 |
| RFC5 | ACGCCCAGAATGCCTTGA | CAACTTTCTCTTCTTCCACGACA | 189 |
| BIRC5 | TTTCTCAAGGACCACCGCA | CAGTGGGGCAGTGGATGAA | 109 |
| PTTG1 | TGGTTGCTAAGGATGGGCTG | GGCTGTTTTTGTTTGAGGGG | 205 |
| PLK1 | CAAGTGGGTGGACTATTCGGA | TCAAGGAGTTGGGATGGGA | 182 |
| DNMT1 | TACCAGGGAGAAGGACAGGG | CACAGACGCCACATCGCC | 149 |
| DNMT3A | TAAGCTGGAGCTGCAGGAGT | GGA AACCAA ATACCCTTTCCA | 179 |
| DNMT3B | ACCACCTGCTGA ATTACTCACGC | GATGGCATCAATCATCACTGGATT | 146 |
| COQ3 | TGACCTGAGGGTGCCATT | AACTGAAGCCCCAAGCC | 151 |
| DOT1 | GAGGCTCTGCGACAAGT | GGAGAAGGGCTCGTAGTT | 181 |
| SUV39H1 | CGATACGGCAATGTGTCTCA | AAAACAATGCTATTCGGGGA | 112 |
| SUV39H2 | TTCACAGTGGATGCGGC | AAAACAATGCTATTCGGGGA | 130 |
| KAT2B | TCTCAACGAAGACTGCGAT | AGTGAAGACCGAGCGAAGC | 162 |
| HDAC5 | TCCTCTATTCCTGGCTGCCT | CACACGTTCACCCGTCACTA | 171 |
| ACTIN | TTA GTT GCG TTA CAC CCT TTC | ACC TTC ACC GTT CCA GTT T | 218 |
